# In Silico Detection of Virulence Genes in Whole-Genome Sequences of Extra-Intestinal Pathogenic *Escherichia coli* (ExPEC) Documented in Countries of the Andean Community

**DOI:** 10.3390/cimb47030169

**Published:** 2025-03-02

**Authors:** Nabila Aldaz, Karen Loaiza, César Marcelo Larrea-Álvarez, Miroslava Anna Šefcová, Marco Larrea-Álvarez

**Affiliations:** 1 Facultad de Ciencias de la Salud, Carrera de Medicina, Universidad Espíritu Santo, Samborondón 092301, Ecuador; 2 Unit of Foodborne Infections, Department of Bacteria, Parasites and Fungi, Statens Serum Institut, 2300 Copenhagen, Denmark

**Keywords:** *Escherichia coli*, ExPEC, virulence factors, whole-genome sequencing, bioinformatic tools, Andean community

## Abstract

*E. coli* pathotypes, which cause extra-intestinal infections, pose significant public health challenges, emphasizing the need for virulence gene surveillance to understand their dynamics. Key virulence genes have been identified in *E. coli* from Andean community countries, predominantly linked to human and animal sources. However, detailed data on virulence profiles from environmental and food sources remain limited. This study utilized an in silico approach to analyze 2402 whole-genome sequences from EnteroBase, known for associations with antimicrobial resistance genes. Of the isolates, 30% were classified as ExPEC, averaging 39 virulence genes per isolate, with adhesin-related genes being the most predominant. These findings were consistent across human, environmental, animal, and food samples. Human and animal isolates exhibited greater diversity in adhesin, secreted factors, and toxin genes compared to other sources, whereas food samples contained the fewest factors. ST449 isolates exhibited an average of 50 virulence genes per genome, with secreted factors and adhesins equally represented, while ST131, ST38, and ST10 carried around 40 genes, predominantly adhesins. Overall, the diversity and frequency of virulence genes exceeded prior reports in the region, highlighting the importance of monitoring these traits to identify emerging patterns in pathogenic *E. coli* strains frequently subjected to antibiotic exposure.

## 1. Introduction

The global rise in multidrug-resistant *Escherichia coli* (MRD *E. coli*) has been extensively documented, with its prevalence varying across geographic regions, countries, and populations [1,2]. This poses a significant threat to public health systems by complicating the treatment of infections caused by pathogenic *E. coli* [3,4]. In South America, key contributors to the dissemination of antibiotic resistance include improper drug use, the spread of genetic traits through the food chain, and environmental pollution [5]. In particular, several strains of *E. coli* resistant to commonly used antibiotics have been identified in countries within the intergovernmental organization known as the Andean community, which includes Colombia, Ecuador, Bolivia, and Peru [6,7,8,9].

*E. coli* is a highly diverse and widely distributed bacterial species present in natural environments. It predominantly resides in the gastrointestinal tract of animals, including humans, where it functions as an essential commensal organism. However, certain *E. coli* strains possess the ability to cause a wide range of diseases, from intestinal to extra-intestinal infections [10,11]. Strains responsible for the latter are classified as extra-intestinal pathogenic *E. coli* (ExPEC) to distinguish them from those associated with intestinal infections (IPEC) or commensal behavior [12]. ExPEC strains can colonize various anatomical sites, leading to the characterization of several pathovars, including uropathogenic *E. coli* (UPEC), septicemic *E. coli* (SePEC), neonatal meningitis-associated *E. coli* (NMEC), and the recently identified endometrial pathogenic *E. coli* (EnPEC). Moreover, *E. coli* associated with avian colibacillosis is classified as avian pathogenic *E. coli* (APEC), which shares mutual virulence traits with ExPEC [13,14,15,16].

Pathogenic and commensal bacteria differ in the repertoire of virulence traits they possess. However, the distinction between fitness factors and virulence factors is often subtle, with some evidence suggesting that the traits associated with ExPEC may have evolved as by-products of a commensal lifestyle [10]. The interaction between ExPEC and the host represents a multifactorial process, incorporating a diverse array of components such as adhesins, regulatory proteins, protective antigens, iron acquisition systems, toxins, invasins, and secreted factors. These elements collectively enable bacteria to colonize, evade the host’s immune response, and establish infection [17,18]. Isolates are conventionally categorized as ExPEC based on the presence of at least two specific genetic determinants, which include *papA* and/or *papC* (encoding P fimbriae), *sfa/focDE* (associated with S and F1C fimbriae), *afa/draBC* (Dr-binding adhesin), *iutA* (aerobactin siderophore system), and *kpsMII* (group 2 capsular polysaccharides) [19].

The Andean community is a regional organization focused on fostering cooperation in various sectors such as industry, agriculture, social affairs, and trade. This continuous exchange of people and resources increases the likelihood of microorganisms spreading across borders and contaminating different environments, particularly those that are highly pathogenic and multidrug-resistant. As previously mentioned, MDR *E. coli* has been reported in Colombia, Ecuador, Peru, and Bolivia [6,7,8,9]. In these countries, the presence of ExPEC has been identified not only in the context of bloodstream and urinary tract infections [20,21,22,23,24], but also in animal cases, including non-human primates, pigs, and bats [25,26,27]. Isolates reported from food and environmental samples have not been conventionally classified as ExPEC. Nonetheless, sequence types (STs) associated with extra-intestinal infections have been recognized in samples from fresh vegetables, ready-to-eat foods, and rivers [28,29,30].

Numerous studies have documented virulence traits in ExPEC, including genes encoding adhesins, iron acquisition components, and protective antigens. These have been identified in human cases from Ecuador [21] and Colombia [22,31] as well as in animal samples from Peru [25,26,27]. Moreover, regulatory factors and toxins have been detected in human isolates from Colombia [22] and in Peruvian animal samples [27]. However, the virulence profiles of food cases from the region remain unexplored, even though evidence shows that food-borne *E. coli* strains can harbor determinants, promoting extra-intestinal infections in humans [17]. Similarly, no studies have assessed virulence genes in environmental samples, although the environment serves as a reservoir for ExPEC strains [32]. Despite the available information on the virulome of ExPEC isolates in the Andean community, it is crucial to expand our current understanding of the interplay between these bacteria, their virulence factors, and their sources of origin.

Whole-genome sequencing (WGS) has enabled the evaluation of the core and accessory genomes of *E. coli* and is now a standard method for isolate profiling. The application of bioinformatic approaches has been crucial in identifying virulence factors, while also helping to uncover the relationships between these factors and highly pathogenic *E. coli* strains [33,34,35]. This study aims to employ VirulenceFinder 2.0 [36] to identify and evaluate the frequency of virulence genes in ExPEC isolates from the Andean community, providing valuable insights into their pathogenic profiles to enhance public health surveillance and One Health initiatives.

## 2. Materials and Methods

### 2.1. Dataset Selection

A dataset of whole-genome sequenced isolates of *E. coli* was created, and sequences were retrieved from the EnteroBase (http://enterobase.warwick.ac.uk/, accessed on 22 March 2023), a platform for studying genomic variation in enterobacteria. The dataset was developed based on the following criteria: (i) reported in countries belonging to the Andean community, Ecuador, Colombia, Bolivia, and Peru; Venezuela was included as it was a member until 2006; (ii) collected between 1993 and 2023; and (iii) non-repetitive whole genomes. A total of 2402 whole-genome sequenced isolates of *E. coli* were retrieved from EnteroBase. Of these, 1031 were associated with human sources, 790 with animals, 534 with the environment, and 47 with food origins. The majority of these sequences were reported in Ecuador (1948), followed by Peru (407), Colombia (24), Bolivia (22), and Venezuela (1).

### 2.2. Identification of Sequence Type (ST) Complexes

To determine the sequence types, the MLST v2.19.0 software developed by Torsten Seemann (Center for Genomic Epidemiology, Technical University of Denmark, Kongens Lyngby, Denmark) was used with default settings (https://github.com/tseemann/mlst; accessed on 22 February 2023). The database available from PubMLST.org was used for the allele analysis scheme [37]. The following genes were utilized for multi-locus sequence typing (MLST) determination: *adk*, *fumC*, *gyrB*, *icd*, *mdh*, *purA*, and *recA.*

### 2.3. Virulence Finder 2.0

Whole-genome sequences were screened for assessment of virulence genes on the Center for Genomic Epidemiology (CGE) (Technical University of Denmark, Kongens Lyngby, Denmark) server using VirulenceFinder 2.0 [36] (https://cge.food.dtu.dk/services/VirulenceFinder/; software version: 2.0.5; accessed on 17 June 2024) with a minimum gene length coverage of 60% and a threshold identity of 90%. VirulenceFinder 2.0 produces an output table with the detected virulence factors followed by the percent identity of the alignment, query length, name, and position in the contig, predicted phenotype, and accession number. An isolate was classified as ExPEC based on the presence of at least two of the following determinants: *papA* and/or *papC*, *sfa/focDE*, *afa/draBC*, *iutA*, and *kpsMII* [19]. Virulence genes were sorted depending on their function into eight groups: adhesins, protective antigens, iron acquisition systems, secreted factors, regulatory proteins, toxins, biofilm formation factors, and invasion factors.

### 2.4. Statistical Analyses

A chi-squared test of independence was performed to compare the frequency of ExPEC isolates, as well as the frequency of virulence factor categories, across different sources and countries. A pairwise comparison was carried out using the Bonferroni correction. Statistical significance was set at *p* < 0.05. These tests were performed in Python (Version 3.9.12) (https://www.python.org; accessed on 22 February 2025; Python Software Foundation, Wilmington, DE, USA). The remaining analyses were carried out in R Studio (Version 2023.09.1+494) (https://www.r-project.org/; accessed on 18 November 2024; Posit, PBC, Boston, MA, USA). For data clustering and heatmap production the Complex Heat map package was utilized [38]. The ratio between a specific gene and the total number of related factors from isolates was determined per source, country, and selected STs.

## 3. Results

### 3.1. Determination of Clonal Complexes and ExPEC Classification

**Figure 1:**
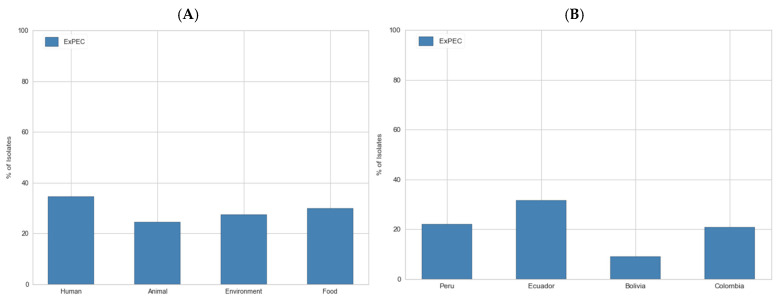
Percentage of ExPEC isolates in the dataset from EnteroBase classified by (**A**) source and (**B**) country.

The selected 2402 genomes belonged to a total of 434 STs (Table S1, Supplementary Results Isolates). Those carrying the *papA* and/or *papC*, *sfa/focDE*, *afa/draBC*, *iutA*, and *kpsMII* genes were classified as ExPEC, totaling 712 isolates, representing 29.6% of the population. When grouped by source, ExPEC accounted for 34% of human, 29% of food, 27% of environmental, and 24% of animal samples. In terms of geographical distribution, ExPEC represented 31% of the isolates in Ecuador, 22% in Peru, 20% in Colombia, and 9% in Bolivia. The frequencies observed in human and animal cases, as well as those in Ecuador and Bolivia, exhibited significant differences (*p* < 0.05) (Figure 1).

Human cases exhibited the highest number of STs (*n* = 80), followed by animal (*n* = 60), environment (*n* = 39), and food samples (*n* = 9). STs were most numerous in isolates reported from Ecuador (*n*= 90), followed by those from Peru (*n*= 38), Colombia (*n*= 4), and Bolivia (*n*= 1). Due to the substantial diversity among isolates, the ten most common STs are listed in Table 1.

### 3.2. Virulence Profile of Extra-Intestinal E. coli Isolates

**Figure 2:**
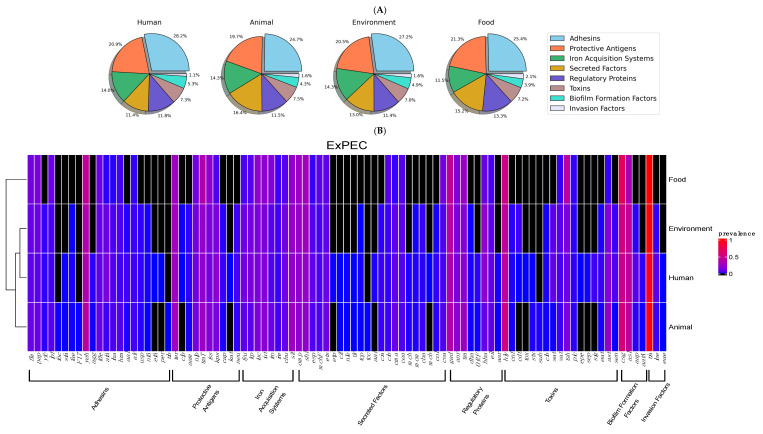
Genes comprising the ExPEC virulome of the whole-genome sequences reported from Andean community countries classified by source. (**A**) Percentages of virulence factors grouped according to their associated function. (**B**) Color heat map denoting the frequency of genes in their category, with blue being the least frequent and red the most abundant; black indicates the absence of genes.

**Figure 3:**
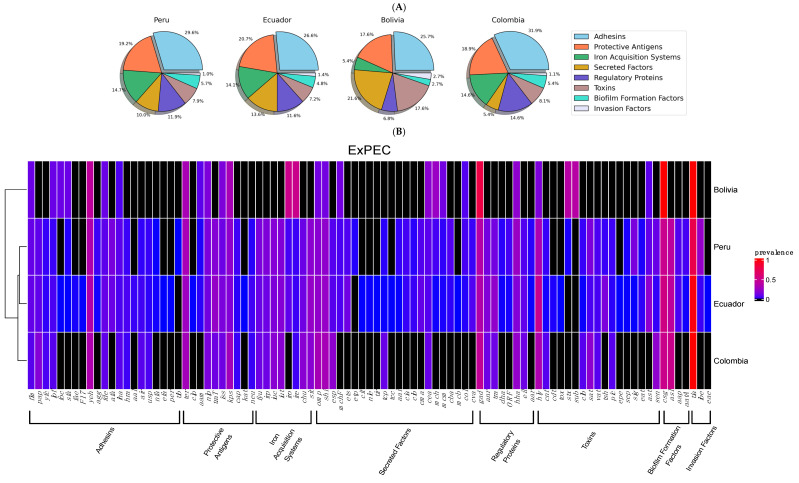
Genes comprising the ExPEC virulome of the whole-genome sequences reported from Andean community countries classified by country of origin. (**A**) Percentages of virulence factors grouped according to their associated function. (**B**) Color heat map representing the frequency of genes within their category, with blue being the least frequent and red the most abundant; black denotes the absence of genes.

A total of 27,543 virulence traits were identified among the 712 samples (Table S2, Supplementary Results Virulence genes), representing an average of 39 genes per isolate. Similar averages were observed when genes were classified by source: human, 39 (13,875/357); environment, 38 (7622/194); animal, 39 (5543/147); and food, 37 (512/14). Ecuadorian isolates exhibited on average 39 factors per isolate, followed by those from Colombia, 37 (185/5); Bolivia, 37 (74/2); and Peru, 36 (3247/90). Genes encoding adhesins and their accessory components were the most abundant, averaging 10 traits per isolate (7436/712). They accounted for approximately 28% of the total number of factors in human and environmental samples, while in food and animal sources, they made up 25% (*p* > 0.05) (Figure 2A). Adhesin genes constituted around 30% of all virulence markers in Colombia and Peru; in Ecuador and Bolivia, they comprised 26% (*p* > 0.05) (Figure 3A).

A total of 21 distinct adhesin genes were identified, with 10 associated with fimbrial adhesins (*fim*, *pap*, *yfc*, *lpf*, *foc*, *sfa*, *fae*, *F17*, *yeh*, and *agg*) and 11 with afimbrial adhesins (*fde*, *afa*, *iha*, *hra*, *aal*, *air*, *usp*, *nfa*, *efa*, *per*, and *tib*). A core set of nine genes was consistently present in all isolates, including the fimbrial genes *yeh*, *fim*, *pap*, and *lpf*, as well as the non-fimbrial genes *fde*, *afa*, *iha*, *hra*, and *air*. The *yeh* alleles were the most abundant, accounting for approximately 35% of all adhesins across the samples. Additionally, the fimbrial genes *ycf* and *fae* along with the non-fimbrial genes *aal*, *usp*, and *nfa*, were detected in isolates from human, animal, and environmental sources. Human and animal isolates also contained the fimbrial genes *sfa* and *agg*, while the afimbrial genes *efa*, and per were found exclusively in human cases. In contrast, the fimbriae encoded by *foc*, and *F17* were solely detected in samples from animal sources (Figure 2B). Isolates from Ecuador contained most of the recorded genes followed by those from Peru, Colombia, and Bolivia (Figure 3B).

Genes encoding protective antigens were the second most common, with around eight factors per isolate (5641/712). They constituted approximately 20% of the population, irrespective of their source of origin (*p* > 0.05) (Figure 2A). Similarly, they accounted for 20% of cases originating from Peru, Ecuador, and Colombia, and 17% of cases from Bolivia (*p* > 0.05) (Figure 3A). A total of 10 distinct gene types were identified, with four associated with environmental stress responses (*ter*, *clp*, *aam*, and *nlp*) and the remaining six linked to immune evasion (*traT*, *iss*, *kps*, and *cap*). Among the stress response genes, those encoding proteins involved in oxidative stress resistance (*ter*), acid resistance (*aam*), and cellular integrity (*nlp*) were present in all isolates. However, the gene encoding a heat shock protein (*clp*) was detected exclusively in environmental and human samples. The immune evasion genes (*traT*, *iss*, *kps*, and *cap*) were consistently present in all isolates; these genes encode proteins involved in immune system inhibition, serum survival, and evasion of phagocytosis through capsule synthesis, respectively. In contrast, the *neu* gene, which is responsible for the synthesis of polysialic acid, was absent in isolates from food samples. Furthermore, the *kat* gene, encoding a catalase-peroxidase enzyme that detoxifies reactive oxygen species (ROS), was identified only in human isolates (Figure 2A). Samples from Ecuador and Peru exhibited greater gene diversity compared to those from Colombia and Bolivia (Figure 3B).

Genes linked to the iron acquisition system averaged five genes per isolate (3889/712). These traits accounted for 14% of all virulence factors in human, environmental, and animal isolates, and 11% in food samples (Figure 2A). They represented 14% of all genes in samples from Peru, Ecuador, and Colombia, but only 5% in Bolivia (Figure 3A). However, no statistical differences were determined (*p* > 0.05). A total of eight distinct genes were identified. The *sit* genes, associated with Fe^2+^ transport, were the most prevalent, along with those involved in the production and transport of aerobactin (*iuc*) and its receptor (*iut*). Genes encoding proteins responsible for the uptake of other siderophores, including enterobactin (*fyu*, *iro*, *ire*) and yersiniabactin (*irp*), were also detected, as was the gene encoding the receptor involved in heme acquisition (*chu*) (Figure 2B). These factors were predominantly associated with samples from Ecuador, Colombia, and Peru, whereas Bolivian cases contained only the *iro* and *ire* genes (Figure 3B).

Secreted factors were present at an average of five traits per isolate (3630/712). The frequencies of the associated genes did not differ across sources (*p* > 0.05) and countries (*p* > 0.05) (Figure 2A) (Figure 3A). A total of 22 distinct genes were identified. Among these, *omp*, *shi*, and *esp*—which are involved in protein degradation and host cell invasion—were among the most prevalent. Moreover, *mchF*, encoding a microcin transporter, and *ets*, associated with the type I secretion system (T1SS), were detected in all genomes. The *cif* and *nle* genes, encoding effectors associated with the type III secretion system (T3SS), were identified in both human and animal isolates. In addition, genes encoding the translocated intimin receptor (*tir*) were also detected in these isolates. In contrast, genes encoding other intimin-associated proteins, such as the Tir domain-containing protein (*tcp*), were found in human, animal, and environmental genomes, while the Tir-cytoskeleton coupling protein (*tcc*) was exclusively observed in animal cases. Traits associated with the type II secretion system (*etp*) and type VI secretion system (*aai*) were exclusively detected in human samples.

Colicin Ib-encoding genes (*cib*) were detected in all isolates, while those linked to colicin Ia (*cia*) were absent in food samples. The *cma* gene, encoding colicin M, and the *cea* gene, encoding colicin E, were present across all genomes. Additionally, the *col* and *cba* genes, responsible for colicin E and B production, were found in human, animal, and environmental cases. All isolates carried the *cva* gene, encoding microcin C, while genes for microcin H (*mch* and *mcm*) were linked to human and animal samples, and the microcin B gene (*mcb*) was found only in human sequences (Figure 2B). Genomes from Ecuador and Peru contained more genes than those from the other two countries, with only the *omp*, *shi*, and *cea* genes present in all cases (Figure 3B).

Regulatory proteins were detected at an average of five genes per isolate (3214/712), representing approximately 14% of the total population in all sources (*p* > 0.05) (Figure 2A). The frequencies of these genes did not differ across countries (*p* > 0.05) (Figure 3A). A total of eight distinct regulatory genes were identified, five of which are associated with adaptation, survival, and metabolism. The *gad*, *anr*, and *tra* genes, encoding proteins involved in responses to low pH, oxygen availability, and conjugative plasmid transfer, respectively, were detected in all genomes. However, the *dha* gene, important for the utilization of dihydroxyacetone as a carbon source, was absent in food samples, and the *ORF* gene, involved in isoprenoid synthesis, was found only in human and animal samples. The remaining three genes are known to regulate virulence and invasion. The *hha* gene, involved in hemolysin expression, and the *eil* gene, associated with invasion and intracellular survival, were found in all samples. The *aar* gene, encoding a regulator of traits related to biofilm formation, was observed only in human and animal genomes (Figure 2B). The *gad* and *hha* genes were present in samples from all countries, while the other genes were mainly detected in Ecuadorian and Peruvian cases (Figure 3B).

Genes associated with toxins averaged three traits per isolate (2011/712). These genes represented around 7% of all virulence genes across all samples (*p* > 0.05) (Figure 2A). Despite the frequency observed in Bolivian isolates, no differences were found with the other genomes (*p* > 0.05) (Figure 3A). A total of 17 distinct genes were identified. Those encoding hemolysins (*hly*), various serine proteases (*vat*, *tsh*, and *pic*), and enterotoxin (*ast*) were present in all isolates. Genes associated with cytotoxic necrotizing factors (*cnf*), genotoxic factors (*clb*), serine protease autotransporters (*sat* and *eat*), and enterotoxin (*sen*) were common but absent in food samples. The *stx* gene, encoding Shiga toxin, and *cdt*, encoding cytolethal distending toxin, were detected exclusively in human and animal isolates and in human and environmental samples, respectively. The *sub* genes were identified only in animal isolates, whereas human genomes exclusively carried genes encoding proteases (*epe*, *sep*, and *sig*) and the *tox* gene, which is responsible for Toxin B involved in cytoskeletal disruption (Figure 2B). These factors were more diverse in isolates from Peru and Ecuador than in those originating from Colombia and Bolivia (Figure 3B).

Biofilm formation traits were evidenced with an average of two genes per isolate (1350/712). Their frequencies were similar among sources (*p* > 0.05) (Figure 2A) and countries (*p* > 0.05) (Figure 3A). Four factors were identified. The *csg* genes, encoding components of curli fibers essential for biofilm formation, and those associated with sulfatases (*asl*) were present in samples from all sources. In contrast, the *app* alleles, which encode dispersin—a protein contributing to biofilm dispersion—were absent in food genomes, and *attA*, involved in dispersin export, was detected only in human and animal samples (Figure 2B). These factors were predominantly found in genomes from Peru and Ecuador. In contrast, Colombian samples contained only *csg* and *asl* genes, while Bolivian samples were positive exclusively for *csg* (Figure 3B).

Invasion factors were detected at an average of less than one gene per isolate (372/712) and represented around 1% across samples from all sources (*p* > 0.05) (Figure 2A) and countries (*p* > 0.05) (Figure 3A). The *tia* gene, associated with the Tia invasion determinant, was identified in isolates from all sources. In contrast, the *ibe* genes, encoding a protein that facilitates the invasion of brain endothelial cells, were absent in food samples. The genes encoding intimin, allowing intimate attachment to host cells, were detected only in human and animal cases (Figure 2B). Samples from Ecuador contained all the aforementioned genes, those from Peru had *tia* and *ibe*, while Bolivian and Colombian isolates carried only *tia* (Figure 3B).

**Figure 4:**
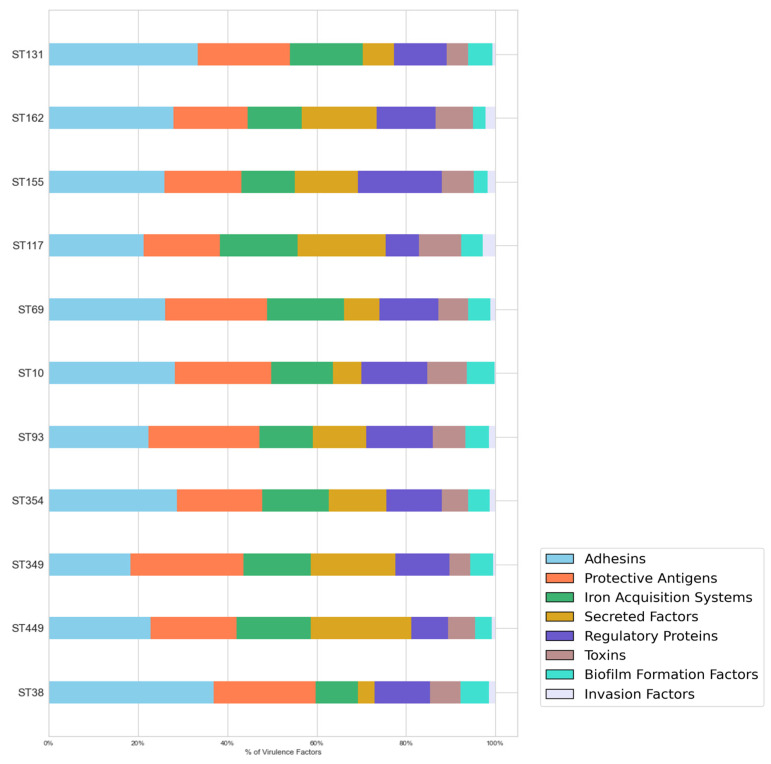
Percentages of virulence factors grouped according to their associated function in ExPEC isolates of the most common STs.

Table 2 presents the average number of virulence genes among isolates corresponding to the most commonly detected sequence types (STs) in the database. Isolates classified as ST449 exhibited an average of 50 or more virulence genes per isolate. In contrast, those identified as ST117, ST354, ST38, ST93, and ST349 had an average of 40 virulence genes, while the remaining STs showed an average of less than 39. ExPEC isolates from these STs were predominantly found in samples from Peru and Ecuador. Figure 4 depicts the distribution of virulence genes among isolates of the selected STs. Overall, adhesin genes were the most common, with more than 30% of the virulence factors in ST38 and ST131 isolates attributed to adhesins. However, in those classified as ST93 and ST349, adhesins ranked as the second and third most common factors, respectively.

Protective antigens accounted for approximately 20% of all virulence factors, being most prevalent in isolates associated with ST349 and ST93. Iron acquisition systems comprised around 14%, with the highest frequency observed in ST117 at 17%. On average, secreted factors represented 13% of virulence genes; however, higher proportions were found in ST449 (22%), ST349 (19%), and ST117 (20%), while lower percentages were observed in ST38 (3%), ST10 (6%), and ST69 (7%). Some genes associated with the T3SS (*cif*, *nle*, *tir*, *tcp*, and *tcc*) were absent across these genomes, whereas the presence of those encoding antimicrobial peptides varied by sequence type (Figure S1). Genes encoding regulatory proteins constituted 12% of all virulence factors. In ST155 and ST93, they accounted for 18% and 15%, respectively, whereas in ST117 and ST449, they comprised only 8%.

Toxin-encoding genes accounted for approximately 7% of all factors. Isolates displayed limited diversity in these genes, with only six distinct types identified. Particularly, only genes encoding hemolysins were present in all genomes (Figure S1). Genes associated with biofilm formation constituted an average of 4% of all virulence factors, with *csg* genes present in all of them. ST38, ST131, and ST10 exhibited a higher number of biofilm-related genes compared to other sequence types (Figure S1). In contrast, genes encoding invasins averaged 1% of the total; in ST117, they accounted for approximately 3%. The *tia* genes were identified in all isolates, while *ibe* were exclusively detected in ST131 and ST354 genomes (Figure S1).

## 4. Discussion

This study investigated the frequency and variety of virulence genes among extra-intestinal *E. coli* isolates identified in Andean community countries. ExPEC isolates constituted only 29% of the total bacterial population examined. Genes encoding adhesins were the most prevalent, followed by those encoding protective antigens, iron acquisition systems, secreted factors, and regulatory proteins, collectively representing approximately 85% of all detected genes. The remaining sequences were associated with toxins, biofilm formation, and invasion factors. Human and animal cases exhibited greater gene diversity compared to environmental and food samples.

ExPEC isolates accounted for 34% of the population in human samples. Various studies have shown the frequency of ExPEC in human cases, particularly those associated with bloodstream and urinary tract infections [20,21,22,23,24]. ExPEC strains have also been identified in animal cases, including non-human primates, pigs, and bats [25,26,27]. We observed that ExPEC isolates accounted for around one-quarter of all isolates across all sources. In the countries within the area of study, isolates reported from these sources have not been classified as ExPEC. However, sequence types (STs) associated with extra-intestinal infections have been documented in samples from the environment and food [28,29,30].

Isolates from STs 117, 69, and 162 were found across all sources. ST117 has been detected in animal cases [25,27], ST69 documented in bloodstream infections [21], and ST162 appeared in food samples [29]. Moreover, the most common sequence types in human, animal, and environmental samples included ST131, ST10, ST38, ST354, ST449, ST349, and ST93. The ST131, ST10, and ST38 clones have been widely studied in the area and are associated not only with urinary tract and bloodstream infections [21,22,39] but also with samples originating from diverse sources, including animals, the environment, and food [25,29,30,40,41,42,43]. Bacteria belonging to ST354 and ST449 have also been documented in Andean community countries [43]. In contrast, ST349 and ST93 have not been reported in the area. The ST155 clone was detected among the most common STs in human, animal, and food samples; however, in environmental cases, this clone was only found in non-ExPEC isolates. ST155 has been reported in the area from diverse sources and has been linked with urinary tract infections [22,41,43].

An average of 39 virulence genes were identified per isolate, with adhesin-encoding genes being the most abundant. Adhesins are essential in bacterial attachment, specificity, and firm binding to host cells. Moreover, they contribute to pathogen invasion, replication, and dissemination, facilitating immune evasion and biofilm formation [18,44]. The *yeh* genes, which encode a group of fimbrial-like adhesins, were the most prevalent of all virulence genes. These proteins facilitate bacterial attachment outside the gastrointestinal tract and are associated with pathotypes responsible for urinary tract infections (UTIs), neonatal meningitis, and sepsis [45,46]. However, these genes have not been documented in isolates reported in the Andean community. Fimbrial-encoding genes, including *fim*, *pap*, *lpf*, and *sfa*, were detected in most genomes and have been previously reported in the context of ExPEC in both human and animal cases [21,22,27,31]. Similarly, afimbrial-encoding genes such as *afa*, *iha*, *usp*, and *air* have been described in the region [21,22,27,31], although we identified additional factors such as *fde* and *hra*, which were consistently present in all cases.

Adhesin genes were followed by those encoding protective antigens, found ubiquitously among samples. These antigens contribute to resistance to oxidative stress, evasion of complement-mediated killing, survival in serum, evasion of phagocytosis, and maintenance of protein homeostasis under stress [17,18]. Genes encoding the enzymes responsible for capsular biosynthesis (*kps*), as well as those for serum resistance (*iss*), complement resistance (*traT*), and tellurite resistance (*ter*), were found in all samples. These genes have been detected in human and animal isolates in Peru and Colombia [22,25,26,27,31]. The stress resistance-associated genes *clp*, *aam*, and *nlp*, along with the immune evasion-related *kat* and *neu* genes described in this study, have not been previously reported in the studied countries.

The iron acquisition system is crucial for scavenging iron from the host, which is essential for bacterial survival and proliferation [47,48]. Eight different genes were detected across all isolates, regardless of the source of origin, with the most common being the *sid*, *iut*, and *iuc* genes. The observed factors have been reported in Peru, Colombia, and Ecuador exclusively in human and animal cases [21,22,25,26,27,31].

Secreted factors contribute to ExPEC colonization, invasion, and persistence within the host by facilitating immune evasion and host tissue damage [17,49]. A total of 22 genes were identified, of which 12 were associated with exported factors and secretion systems (*omp*, *shi*, *esp*, *mchF*, *ets*, *etp*, *cif*, *nle*, *tir*, *tcp*, *tcc*, and *aai*), while the remaining genes encoded bacteriocins (*cia*, *cib*, *cma*, *cea*, *mch*, *mcm*, *cba*, *mcb*, *col*, and *cva*). Bacteriocins, as antimicrobial peptides, eliminate competing microorganisms, allowing ExPEC to colonize and dominate specific host niches [50,51]. The *omp* genes have been previously reported in urinary tract infections (UTI) in Colombia [22,31] and animal samples from Peru [26]. The presence of *mchF*, *mcm*, and *cva* genes has also been documented in Peruvian animal isolates [27,40]. However, the remaining factors have not been described in the region.

Regulatory proteins are crucial for coordinating the complex responses required to adapt to different environments and host conditions. These factors enable ExPEC to colonize, invade, and survive more effectively, thereby leading to infections [17,18]. Genes encoding glutamate decarboxylase (*gad*) were the most prevalent. Also, other genes associated with adaptation, survival, and metabolism (*anr*, *tra*, and *dha*), as well as those involved in virulence regulation (*hha*, *eil*, and *aar*), were detected in the majority of samples. Only the *gad* and *eil* genes have been previously described in samples from the area [22,27].

Toxins contribute to the pathogenesis of ExPEC by disrupting cell membranes, which leads to cell lysis and tissue damage. They also modulate the host immune response, either by directly killing immune cells or by altering immune signaling pathways [18,52]. Hemolysin-encoding genes (*hly*) were detected across all samples, along with genes encoding autotransporter serine proteases, including *vat*, *tsh*, *pic*, and *ast*. The *pic*, *tsh*, and *ast* genes have been previously reported in *E. coli* isolates from animal samples in Peru [25,27], while *hly* has been associated with bacteria causing UTIs in Colombia [22]. In addition, Colombian isolates have demonstrated the presence of the *sat* and *sen* genes [22], both of which were detected in our analysis, not only in human samples but also in genomes from animals and the environment. The remaining identified traits have not been previously described in the countries under study.

Biofilm formation factors support the synthesis of biofilms, which protect bacteria from host immune defenses, enhance antibiotic resistance, support bacterial persistence during chronic infections, and facilitate the coordination of other virulence factors [17,18]. The *csg* and *asl* genes were the most common and the only ones associated with food cases. The other identified genes have not been described in isolates from the area of study (*ibe*, *tia*, and *eae*). The *ibe* genes facilitate the invasion of brain endothelial cells, enabling ExPEC to breach the blood–brain barrier and cause meningitis. The *tia* genes promote the invasion of epithelial cells, supporting colonization and the establishment of infections [53,54]. None of these factors have been described in isolates from Andean community countries. The *eae* genes, encoding intimin—a protein typically associated with enteropathogenic *E. coli* (EPEC) and known to disrupt brush-border microvilli [55] —were detected in both human and animal samples. These sequences have been reported in the context of (IPEC) linked to food sources in Colombia [56]. However, evidence suggests that *eae*-containing isolates may also harbor ExPEC-associated determinants, including *traT*, *iss*, and *iutA* [57,58].

ExPEC associated with the ST449 clone showed an average of 50 genes per isolate. Furthermore, in these genomes, secreted factors were as prevalent as adhesins, including sequences for secreted effectors (*omp* and *esp*) and bacteriocins (*cma*, *cea*, *cba*, and *col*). ST449 has been reported in the area and is associated with class 1 integrons and their linked antibiotic-resistance genes [43]. This clone has also been related to extended-spectrum β-lactamases in *E. coli* populations colonizing hospital patients [59]. *E. coli* isolates belonging to the diarrhoeagenic enteroaggregative pathotype have been classified as ST449 [60], although no reports were found linking this clone to ExPEC infections nor reports detailing the associated virulence markers. Isolates grouped as ST131, ST38, ST10, ST117, ST354, ST93, and ST349 exhibited an average of 40 virulence genes. ST131 and ST38 had the highest percentage of adhesin-encoding genes, and these clones, along with ST10, also demonstrated greater diversity in these genes compared to other relevant STs. Adhesin-encoding genes, such as *fim*, *pap*, *sfa*, *iha*, and *afa*, have been reported in isolates belonging to these sequence types in Andean community countries [21,22,31].

In ST131 isolates, the *usp* genes have also been found, while in ST10 and ST117 isolates, the *lpf*, *air*, and *iha* adhesin genes have been described [22,27,31]. However, the present results indicate the occurrence of other adhesin genes, including the *yeh* and *fde* alleles. It has been shown that isolates from the above-mentioned STs carry genes associated with regulatory proteins such as *gad* (ST131, ST10, ST38, and ST117) and *eil* (ST10) [22,27], as well as protective antigens such as *kps*, *iss*, *cap*, *traT* (ST131, ST10, ST38, and ST117), and *ter* (ST10) [21,22,25,26,27,31]. Genes encoding components of the iron acquisition system have also been identified, including *iut* (ST131, ST10, ST38, and ST117), *fyu* (ST131, ST10), *iuc*, *sit*, *irp* (ST10), *ire*, and *iro* (ST10 and ST117) [21,22,25,26,27,31]. In the ST131 and ST38 clones, the occurrence of the *hly*, *sat*, and *sen* toxins has been noted, while *ast* and *pic* have been determined in ST117 and ST10. In the latter sequence type, alleles of the *mcm* and *tsh* genes, as well as the *cva* genes, have also been highlighted [21,22,25,26,27,31]. The present outcomes show that the genes associated with the aforementioned traits were highly prevalent across all samples and STs, with higher diversity than previously reported in the literature. Furthermore, this in silico analysis revealed that the ExPEC isolates related to these STs bore genes of other virulence categories, such as biofilm formation factors (*csg* and *asl*) and invasins (*tia* and *ibe*).

ST354 has been linked to extra-intestinal infections, and some virulence genes have been described, such as those encoding adhesins, protective antigens, toxins, the iron acquisition system, and invasins [61,62]. Samples grouped under this sequence type showed the observed distribution of virulence genes, adhesins were the most prevalent, and particularly gene diversity was limited regarding toxin-encoding genes. The ST354 has been documented in samples from Andean community countries and has been associated with multidrug resistance and the presence of class 1 integrons, although no references to virulence markers have been made [29,43]. As previously mentioned, there are no reports regarding ExPEC cases linked to ST93 and ST349 in the area. These clones exhibited the overall distribution of virulence genes.

A core set of shared virulence factors was identified in ExPEC isolates, which were also the most prevalent across the studied reservoirs. The high frequency of adhesins, protective antigens, iron acquisition systems, and secreted factors in non-human sources suggests a potential risk for foodborne and environmental contamination, as well as zoonotic transmission. While human samples exhibited greater gene diversity, a similar distribution and frequency of the most common virulence factors—including regulatory proteins, toxins, biofilm formation, and invasion factors—were observed across human, animal, and environmental isolates. The presence of highly virulent strains in these sources raises concerns regarding their potential dissemination through trade, travel, or agricultural practices. Tracking virulence patterns is crucial for public health, as it facilitates the identification of novel clones with pathogenic potential, aiding in early detection and response efforts. For instance, reports on the presence and virulence profiles of ST349 and ST93 are scarce; yet, these STs exhibited an average number of virulence genes comparable to well-characterized STs in the region, such as ST131, ST10, and ST38. Furthermore, isolates classified as ST449 carried a higher-than-average number of virulence genes, predominantly encoding adhesins and secreted factors. This finding suggests their potential for widespread dissemination, particularly given that ST449 has been linked to the presence of resistance integrons in the region. Consequently, integrating virulence profiling into surveillance programs is essential for improving the monitoring of ExPEC-related infections and informing prevention strategies and biosecurity measures to mitigate their spread.

The use of the EnteroBase to construct the dataset of *E. coli* sequences in this study might introduce a potential bias towards culturable or pathogenic bacteria. Although new isolates will continue to be documented, the ones analyzed in this study represent significant examples. Furthermore, this investigation employs a database of well-characterized marker genes, which may account for only a partial representation of the population. Despite the acknowledged limitations, these findings enhance our understanding of the associations between virulence genes and extra-intestinal pathogenic *E. coli* isolated from various sources in Andean community countries.

## 5. Conclusions

Almost one-third of the isolates were classified as ExPEC and were identified in samples from humans, animals, the environment, and food, with an average of virulence genes per isolate of approximately 39 across all categories. The majority of genes encoding protective antigens, iron acquisition systems, and regulatory proteins were consistently detected across all samples, exhibiting similar frequency. In contrast, genes associated with adhesins, secreted factors, and toxins demonstrated greater diversity in human and animal cases compared to those derived from environmental and food sources. Adhesin genes were the most frequent, constituting approximately one-quarter of all virulence genes, with *yeh* alleles being the most common. In addition, genes associated with biofilm formation and invasion factors were identified in all sample types, with greater diversity observed in human and animal cases. Isolates of the ST449 clone averaged 50 factors per genome, with toxin-encoding genes being as prevalent as adhesins. Similarly, isolates from ST131, ST38, and ST10 averaged 40 genes, with a significant proportion encoding adhesins. Overall, these findings demonstrate a broader abundance and diversity of virulence traits across all sample types and STs compared to earlier studies. Monitoring the prevalence and diversity of these traits is therefore essential. In silico analyses assist in identifying virulence factors and detecting emerging patterns of pathogenicity, particularly in *E. coli* strains responsible for extra-intestinal infections that are frequently exposed to antibiotic pressure. Future research should explore comparative genomics, surveillance modeling, and functional analysis to identify unique virulence traits, elucidate their mechanisms, and enhance outbreak prediction, ultimately strengthening public health strategies to control ExPEC spread in the region.

## Figures and Tables

**Table 1:** Top ten most frequently detected sequence types (STs) among ExPEC isolates, categorized by source and country of origin.

Rank	Human (*n* = 357)		Animal (*n* = 194)		Environment (*n* = 147)		Food (*n* = 14)	
	ST	*n* * (%)	ST	*n* (%)	ST	*n* (%)	ST	*n* (%)
1	38	43 (12)	354	21 (11)	117	21 (14)	224	5 (36)
2	10	39 (11)	162	19 (10)	38	17 (12)	973	2 (14)
3	354	23 (6)	117	16 (8)	131	15 (10)	155	1 (7)
4	131	22 (6)	155	10 (5)	224	10 (7)	117	1 (7)
5	117	15 (4)	449	9 (5)	449	9 (6)	162	1 (7)
6	93	15 (4)	38	7 (4)	10	8 (5)	69	1 (7)
7	449	14 (4)	453	7 (4)	1193	7 (5)	12,486	1 (7)
8	349	11 (3)	48	7 (4)	69	6 (4)	4204	1 (7)
9	162	11 (3)	349	7 (4)	162	6 (4)	847	1 (7)
10	69	9 (3)	93	6 (3)	354	5 (3)		
**Rank**	**Peru ** **(*n* = 90)**		**Ecuador ** **(*n* = 615)**		**Colombia ** **(*n* = 5)**		**Bolivia ** **(*n* = 2)**	
	**ST**	***n* (%)**	**ST**	***n* (%)**	**ST**	***n* (%)**	**ST**	***n* (%)**
1	10	23 (26)	38	65 (11)	69	2 (40)	442	2 (100)
2	452	5 (6)	117	52 (8)	59	1 (20)		
3	349	4 (4)	354	47 (8)	354	1 (20)		
4	127	4 (4)	162	37 (6)	73	1 (20)		
5	155	4 (4)	131	35 (6)				
6	131	3 (3)	449	30 (5)				
7	93	3 (3)	10	28 (5)				
8	12517	3 (3)	224	22 (4)				
9	449	2 (2)	93	22 (4)				
10	57	2 (2)	349	17 (3)				

* Number of isolates.

**Table 2:** Average number of virulence genes in ExPEC isolates of common STs by source and country of origin.

Rank	Animal (*n* = 194)				Environment (*n* = 147)				Human (*n* = 357)				Food (*n* = 14)			
	ST	Virulence Genes	*n* *	avg.	ST	Virulence Genes	n	avg.	ST	Virulence Genes	*n*	avg.	ST	Virulence Genes	*n*	avg.
1	449	484	9	54	449	454	9	50	449	737	14	53	4204	47	1	47
2	117	706	16	44	69	256	6	43	117	620	15	41	12486	46	1	46
3	38	302	7	43	224	402	10	40	349	453	11	41	117	42	1	42
4	354	892	21	42	354	197	5	39	38	1741	43	40	162	40	1	40
5	93	239	6	40	38	646	17	38	354	926	23	40	224	184	5	37
6	349	269	7	38	117	783	21	37	93	603	15	40	155	36	1	36
7	453	267	7	38	131	558	15	37	69	353	9	39	973	60	2	30
8	48	250	7	36	162	216	6	36	131	821	22	37	847	29	1	29
9	162	675	19	36	10	273	8	34	10	1392	39	36	69	28	1	28
10	155	311	10	31	1193	224	7	32	162	376	11	34				
**Rank**	**Peru** **(*n* = 90)**				**Ecuador** **(*n* = 615)**				**Colombia** **(*n* = 5)**				**Bolivia** **(*n* = 2)**			
	**ST**	**Virulence Genes**	***n***	**avg.**	**ST**	**Virulence Genes**	***n***	**avg.**	**ST**	**Virulence Genes**	***n***	**avg.**	**ST**	**Virulence Genes**	**n**	**avg.**
1	449	106	2	53	449	1569	30	52	73	45	1	45	442	74	2	37
2	57	94	2	47	354	1949	47	41	59	44	1	44				
3	93	118	3	39	117	2110	52	41	69	66	2	33				
4	127	148	4	37	38	2621	65	40	354	30	1	30				
5	10	794	23	35	349	684	17	40								
6	131	103	3	34	93	852	22	39								
7	349	136	4	34	131	1318	35	38								
8	452	163	5	33	224	822	22	37								
9	12,517	93	3	31	10	1009	28	36								
10	155	108	4	27	162	1307	37	35								

* Number of isolates.

## Data Availability

The data are available upon request.

## Supplementary Materials

The following supporting information can be downloaded at https://www.mdpi.com/article/10.3390/cimb47030169/s1. Table S1: Supplementary Results Isolates; Table S2: Supplementary Results Virulence genes; Figure S1: Genes comprising the virulome of ExPEC isolates belonging to the most common STs.
