# Supplementary material for: In Silico Detection of Virulence Genes in Whole-Genome Sequences of Extra-Intestinal Pathogenic Escherichia coli (ExPEC) Documented in Countries of the Andean Community
Source: Curr Issues Mol Biol. 2025 Mar 2;47(3):169. doi: 10.3390/cimb47030169 (PMC11941574; doi:10.3390/cimb47030169)
Supplement: Supplementary file 1 [file Figure S1. Genes comprising the virulome of ExPEC isolates belonging to the most common STs.pdf › Figure S1. Genes comprising the virulome of ExPEC isolates belonging to the most common STs.pdf]

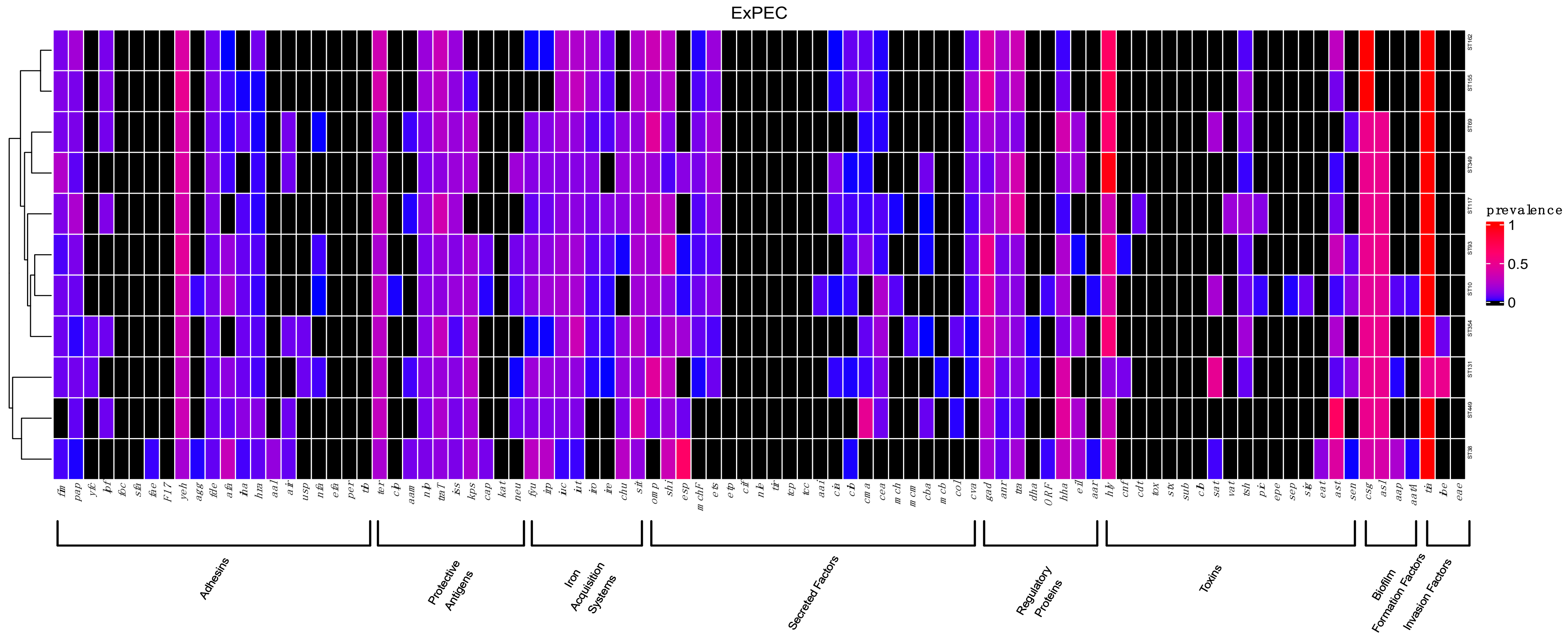

Figure S1. Genes comprising the virulome of ExPEC isolates belonging to the most common STs. Color heat map representing the prevalence of genes within their category, with blue being the least frequent and red the most abundant; black denotes the absence of genes.
